# Lipofibromatosis: A Rare Diagnosis on Fine Needle Aspiration Cytology

**DOI:** 10.5146/tjpath.2020.01479

**Published:** 2020-09-15

**Authors:** Arti Khatri, Nidhi Mahajan, Mamta Sengar, Anil Agarwal

**Affiliations:** Departments of Pathology, Chacha Nehru Bal Chikitsalaya, Geeta Colony, Delhi, India; Departments of Pediatric Surgery, Chacha Nehru Bal Chikitsalaya, Geeta Colony, Delhi, India; Departments of Orthopaedics, Chacha Nehru Bal Chikitsalaya, Geeta Colony, Delhi, India

**Keywords:** Lipofibromatosis, Cytology, Pediatric, Recurrence

## Abstract

Lipofibromatosis is a recently recognized slow growing rare pediatric tumor. Paucity of its cytological description in the literature leads to its pre operative misdiagnosis and further incomplete management. A twelve-month-old female presented with a rapidly progressive mass in the right thigh and buttock region. On examination, the mass was huge and involved the medial, posterior and lateral aspects of the thigh. The cytological smears showed mature adipocytes with few spindled out cells. FNA was reported as a lipoma, corroborating with the radiological presumptive diagnosis. However, histopathological and immunohistochemical features favoured a diagnosis of Lipofibromatosis. The cytological smears were reviewed and a cyto-histo correlation was established. The diagnosis of Lipofibromatosis rests upon classical cytological features in a clinically and radiologically suggestive picture. An early and accurate diagnosis if established can help the surgeon plan excision with wider margins as incomplete excision is associated with a high rate of recurrence.

## INTRODUCTION

Lipofibromatosis (LF) is an uncommon pediatric soft tissue tumour, more often seen in males (M: F -2.7:1) (1). It is usually seen from birth to childhood and presents as a subcutaneous mass upto 5 cms in size (2). The lesion is known for its recurrence if excised incompletely, but no metastasis however has been reported so far (1). Due to the rarity of the tumor, there is paucity of its cytological description in the literature. We hereby present a twelve-month-old female child with a huge right gluteal and thigh mass, together with the radiology, cytology and histopathological correlation.

## CASE REPORT

**Figure 1:**
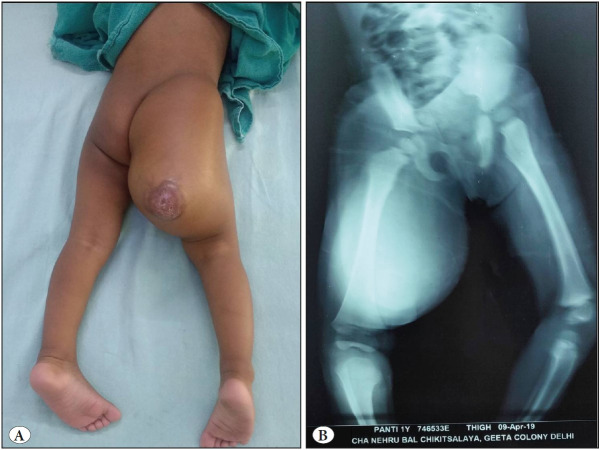
**A)** Clinical picture of the right gluteal and thigh mass with an area of skin ulceration. **B)** Radiograph shows a large radiolucent mass. Underlying bone is not involved.

A twelve-month-old girl presented with a rapidly progressive painless mass over the right gluteal and upper thigh region. The mass was seen at birth in the right buttock and was of the size of a lemon to begin with; however, it had rapidly grown over the last six months and now extended over the thigh. The patient gave a history of incision and drainage of that site in a private hospital in view of it being reported as a gluteal abscess with mild vascularity on Ultrasound. On examination, the mass was diffusely involving the medial, posterior and lateral aspect of the right upper thigh extending into the gluteal region, measuring 17 x 14 cm with an ulcer on the overlying skin (Figure 1). No thrill or bruit was palpated. The mass was firm to hard, immobile and non-tender. X-Ray (Figure 1) and CT scan of the mass were acquired, both of which suggested a poorly circumscribed thigh mass deep to the muscles suggestive of an adipocytic lesion, possibly lipoma.

**Figure 2:**
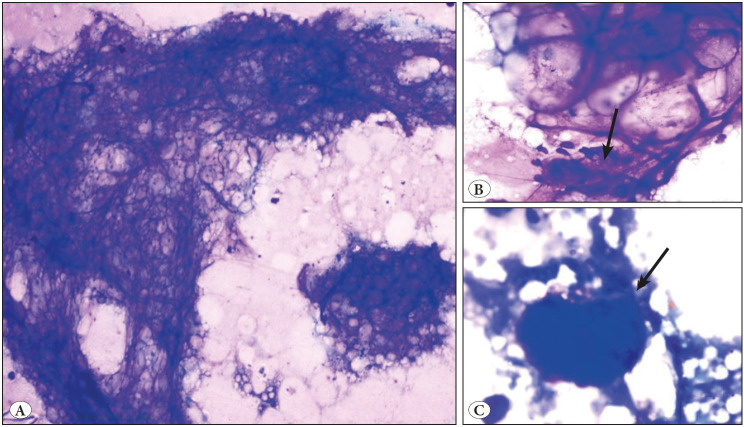
**A)** Cytology smears showing moderate cellularity comprising large mature adipose tissue fragments (Giemsa; x100). **B)** Smears showing bland spindle cell cluster closely associated with the adipocytes (black arrow) (Giemsa; x400). **C)** Showing a viable muscle fragment in a lipidaceous background (black arrow) (Giemsa; x400).

**Figure 3:**
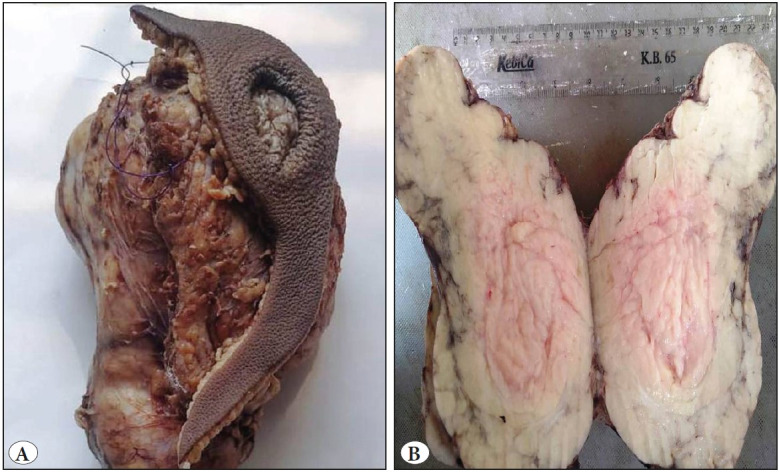
**A)** Gross specimen showing a well delineated mass with an ulcer in the overlying skin. **B)** Cut section is yellow, lobulated and solid.

**Figure 4:**
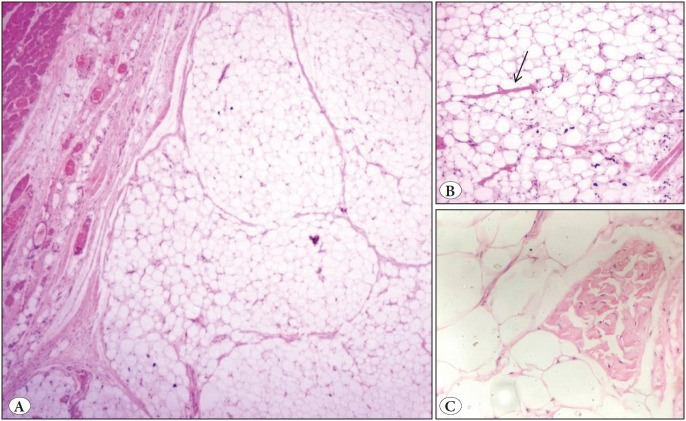
**A)** Microscopic section showing lobules of mature adipose tissue (H&E; x100). **B)** Section showing benign appearing spindled out cells and skeletal muscle interspersed within these mature adipocytes (black arrow) (H&E; x100). **C)** Section showing high power view of the skeletal muscle bundles (H&E; x100).

Fine needle aspiration cytology (FNAC) was done and showed a moderately cellular lesion composed of large fragments of mature adipose tissue admixed with singly scattered and groups of plump to spindle cells (Figure 2). No lipoblast was seen. No nuclear pleomorphism, mitosis or necrosis was identified. In view of the cytological features, possibility of a benign lesion of adipocytic origin (lipoma) was suggested and an excision for histopathological correlation was advised in view of the large size of the swelling for further complete evaluation. Per operatively, it was an ill- defined solid mass deep to the thigh and right gluteal muscle but could be easily separated from the sciatic nerve. Grossly, the mass was grey white, measuring 17 x 13 x 12 cm and partly covered by an elliptical skin flap which showed a central ulceration (Figure 3). The cut surface was yellow and lobulated with no areas of haemorrhage and necrosis (Figure 3). Microscopic examination revealed a well delineated lesion consisting of lobules of mature adipose tissue with interlacing fibrous bands of variable thickness (Figure 4). The bands comprised bland looking fibroblasts with minimal nuclear pleomorphism. Interspersed in between the mature adipose tissue were few lipoblast-like cells. No true lipoblast was seen. The lesion also showed few entrapped skeletal muscle bundles (Figure 4). No mitotic figure/necrosis was seen. The mature adipose tissue was seen to infiltrate the adjacent skeletal muscle. The overlying skin and skin resected margins were free of the lesion, with the lesion almost reaching up to the deep resected plane. The lesion showed negative staining for Desmin and PLAG-1 on immunohistochemistry. The diagnosis of LF was made. Cytology slides were reviewed and cyto-histo correlation was established. Thus presence of large fragments of adipose tissue, viable muscle fragments in close proximity to the adipocytes (Figure 2, inset), and spindled out cells on cytological smears warranted a diagnosis of LF. The patient did well post operatively and there is no recurrence so far (follow up of 5 months).

## DISCUSSION

Lipofibromatosis was first described in the year 2000 by Fetsch et al. in a series of 45 cases, which were previously reported as infantile or juvenile fibromatosis of the non-desmoid type (1). This tumor presents as a slow growing ill-defined subcutaneous mass in the extremities, trunk, back and head and neck region of children, usually not larger than 5 cm (3). Deepti AN et al. reported LF in the foot with size of 10 cm (2). A case of diffuse upper limb involvement has been reported in a two-day-old male child but the size of which has not been mentioned (4).

The lesion is known to occur from birth to early second decade of life but can also be seen congenitally. Though the median age of presentation in the two large series is 1 year, the majority of the cases usually present by 3 years of age and 18% of these lesions are congenital (3). It is more commonly seen in boys with a male to female ratio of 2.7:1 and the majority of the case reports are from male patients (1). Two large series of LF have been published, one with six cases, of which two were females, and the other with 20 cases, of which six were females (2,3). Our case was a female and presented congenitally. Also, it is the largest LF operated on and reported so far in the literature.

The etiopathogenesis of LF is still unclear. No recurrent genetic alteration has been detected. Al-Ibraheemi et al. in their study on 20 cases of LF found recurrent FN1-EGF gene fusion in four of their cases. This genetic aberration was found to be characteristic feature of calcifying aponeurotic fibroma. Based on this, they suggested that some cases of LF actually represent early stages of calcifying aponeurotic fibroma with no calcification. Their study found 8 other genetic fusions which are known to activate the PI3K-AKT-mTOR pathway (5). A single case report of LF has also been reported with multiple congenital anomalies such as trigonocephaly, cleft lip and palate and syndactyly (6). Our case had no other congenital anomaly. Molecular studies were not performed as they were unavailable.

Radiological imaging can help distinguish LF from other entities, though not always diagnostic. Ultrasound is usually nonspecific. MRI is the imaging modality of choice, which shows LF as a well-defined mass with hyperintense signalling that is isointense to fat on T1- and T2- weighted images. These signals however vary depending upon the proportion of adipocytic and fibroblastic components. Hence, MRI is only suggestive and therefore partially helps in pre-operative planning. The cytological features of this entity are not reported often in the literature and the definitive diagnosis rests upon histopathology. The tumor is associated with high recurrence rates of about 72% (1). Boss et al. reported a recurrence of 33% in its series of six cases of LF (3). No metastasis however has been reported so far. Thus, a pre-operative cytological diagnosis is essential for management of these cases as complete surgical excision with a wide margin ensures low recurrence.

The cytological indicators of LF are presence of skeletal muscle fibres, mature adipose tissue and benign appearing spindle cells clusters in close proximation. The background may be lipidaceous or myxoid. A few stromal fragments may also be noted. No true lipoblast is seen. There is absence of nuclear pleomorphism, necrosis and mitosis. Grossly, LF is unencapsulated with poorly defined margins. The cut section is yellow with whitish streaks. The microscopic sections show abundance of mature adipose tissue (> 50%) traversed by a fibroblastic component. The latter is comprised of bland spindle shaped cells and sometimes may show myxoid change. Small univacuolated cells, which are actually degenerating adipocytes or lipid rich fibroblasts, are seen at the interface of fibroblast and adipose tissue. These may be mistaken for lipoblasts. No nuclear atypia or necrosis is seen. Mitotic figures were not seen in our case but have been reported by Fetsch et al. in one third of the cases (1,4,6). Immunohistochemistry is neither specific nor required. However, the cells may express CD 99, BCL 2, S-100, CD 34 and Actin. LF can be differentiated from other adipocytic, myofibroblastic and fibroblastic lesions of childhood clinically, cytologically and histopathologically (Table 1) (7–10). This differentiation is important in view of the significantly different prognosis and management. When incompletely excised, this tumor has a high rate of local recurrence which may be destructive or obstructive depending upon its site, making complete surgical removal with wide margins imperative. Metastasis has not been reported so far, though factors like congenital onset, male gender and mitotic activity in the fibroblastic component have been associated with recurrence (1).

**Table 1:** Characteristics of the differential diagnosis of Lipofibromatosis.

**Differential Diagnosis**	**Age**	**Sex**	**Size**	**Location**	**Progression**	**Imaging**	**Cytology**	**Gross**	**Histopathology**	**Recurrence**
Lipoblastoma	< 3 yrs	M>F	4.5 to 21 cm (Mean 10.8cm)	Extremities> trunk. Majority subcutaneous 75% left sided	Rapid progression, 8q11-13 abnormalities	Well circumscribed, Low density images on T1-weighted MRI due to increased cellularity	Moderate to poorly cellular Clusters of adipocytes, lipoblasts, spindle cells Background -myxoid	Well circumscribed, encapsulated. Cut surface - yellow, myxoid, small cystic foci +	Lobules of lipoblasts with mature adipose tissue separated by cellular fibrous septae, plexiform capillary network & abundant myxoid stroma.	80%
Lipoblastomatosis	< 3 yrs	M>F	Variable	Extremities, Diffusse & deep seated	Rapid Progression	Poorly circumscribed, low density of T1 images	Diffuse, unencapsulated, can infiltrate into adjacent soft tissue	Poorly circumscribed, pale yellow cut surface, less lobular appearance	Infiltrates skeletal muscle & less lobulated	Common
Fibrolipomatous hamartoma of nerve/ Fibrolipoma of nerve/ macrodystrophia lipomatosa	1st- 4th decade	M:F 1:2 with macrodactyly & 1:1 ratio in cases without.	2-12 cm	85% median nerve and its digital branches (hand, wrist and forearm)	Rarely progresses	MRI reveals a hypodense lesion evenly distributed between nerve bundles (cable-like appearance coaxially)	Clusters of spindle shaped cells with wavy nuclei, adipose tissue & stromal fragments	Fusiform enlargement of nerve by yellow adipose tissue, confined within epineurium	Infiltration of epineurium and perineurium by adipose and fibrous tissue (collagen), causing enlargement of nerve. Concentric perineurial fibrous tissue and pseudo-onion bulb formation. Occasionally metaplastic bone	33 - 60%
Calcifying aponeurotic fibroma/ Juvenile nodular aponeurotic fibroma/ Keasbey tumor	8-14 years	M>F	Usually < 3 cm	Extremities	Slow progression	CT shows an ill-defined tumorwith variable fine, stippled calcifications	Benign appearing spindle cells, chondroid cells, multinucleated giant cells, calcified debris +	Nodular gray-white, gritty mass in subcutaneous tissue or tendon	Cellular nodules of epithelioid cells with palisading chondroid differentiation and calcification Usually lacks adipose tissue component	50%
Intramuscular lipoma/ invasive/ infiltrative lipoma	4th -7th Decade	F>M	1 - 25 cm	Thigh, trunk, head and neck	Slow progression	CT- hypodense mass situated within the muscle.	Adipose tissue admixed with viable skeletal muscle bundles	Poorly circumscribed, variably ecapsulated, extends between and around skeletal muscle fibers	Mature adipose tissue infiltrates into the skeletal muscle	20%
Spindle cell Lipoma	4th -7th Decade	M> F	< 10 cm	Neck, back or shoulder	Slow progression	T1-W images show isointensity of non adipose components to skeletal muscle & variable signals on T2-W sequences.	Scattered & clusters of mildly pleomorphic spindle cells admixed with mature adipocytes, mast cells and small stromal fragments	Well circumscribed, cut surface- yellowish to whitish	Mixture of mature adipocytes and bland spindle cells in mucinous / myxoid/fibrous background with ropey collagen.	Rare
Infantile myofibromatosis/ Myofibroma	1st- 2nd decade	Solitary M>F Multicentric F>M	1 to 3 cm	Head, neck and trunk	Variable progression	Well circumscribed bone lucency	Variable cellularity, spindle-shaped fibroblasts in groups interspersed with stromal fragments	Well circumscribed, uncapsulated, deeper lesions are more infiltrative and diffuse	Nodules of spindle cells in myxoid background with slit-like vascular spaces, Vimentin, SMA +	Spontaneous resolution, Good prognosis Visceral involvement- poor prognosis (MR- 70%)
Hemosiderotic Fibrohistiocytic Lipomatous Lesion/ Hemosiderotic Fibrolipomatous Tumor	4th- 6th Decade	F>M	< 20 cm	Distal extremities	Slow progression	Isointense to fat on T1- and T2-W sequences,	Mature adipose tissue with pleomorphic spindled cells, scattered inflammatory cells, abundant hemosiderin pigment	Unencapsulated but circumscribed, Cut surface, darker yellow than surrounding fat.	Lobules of fat separated by fibrous septae, spindle cells in the fibrous septa infiltrate into fat producing a honey comb pattern. Abundant hemosiderin primarily in histiocytes in the spindled area. No significant atypia/mitosis	High
Fibrous Hamartoma of infancy	< 2 years	M>F	<5 cm	Axilla, shoulder & inguinal region	Rapid progression	Heterogeneous hyperechogenicity with a “serpentine pattern” of intervening hypoechoic portions.	Fragments of fat cells mixed with bland spindle cells with or without primitive mesenchymal cells	Poorly circumscribed, gray-white with yellow fat	Triphasic pattern of fibroblasts or myofibroblasts, islands of mature fat and primitive spindle cells	Rare

Our experience with the current case has led to the conclusion that Lipofibromatosis may be congenital, can occur in females and can present as a large mass involving the extremities. The diagnosis can be made with near precision on FNAC if classical cytological features are present along with clinical and radiological suspicion. A pre-operative diagnosis of this rare entity can help the surgeon plan complete resection with wider margins to prevent future recurrence.
